# Two-Qubit Local Fisher Information Correlation beyond Entanglement in a Nonlinear Generalized Cavity with an Intrinsic Decoherence

**DOI:** 10.3390/e23030311

**Published:** 2021-03-06

**Authors:** A.-B. A. Mohamed, E. M. Khalil, M. F. Yassen, H. Eleuch

**Affiliations:** 1 Department of Mathematics, College of Science and Humanities in Al-Aflaj, Prince Sattam Bin Abdulaziz University, Al-Aflaj 11942, Saudi Arabia; mf.ali@psau.edu.sa; 2 Faculty of Science, Assiut University, Assiut 71515, Egypt; 3 Department of Mathematics, College of Science, Taif University, P.O. Box 11099, Taif 21944, Saudi Arabia; eiedkhalil@tu.edu.sa; 4 Department of Mathematics, Faculty of Science, Damietta University, Damietta 34511, Egypt; 5 Department of Applied Physics and Astronomy, University of Sharjah, Sharjah 27272, United Arab Emirates; hichem.eleuch@adu.ac.ae; 6 Department of Applied Sciences and Mathematics, College of Arts and Sciences, Abu Dhabi University, Abu Dhabi 59911, United Arab Emirates; 7 Institute for Quantum Science and Engineering, Texas A&M University, College Station, TX 77843, USA

**Keywords:** nonclassical correlation, intrinsic decoherence SU(1,1), SU(2)-algebraic treatment

## Abstract

In this paper, we study a Hamiltonian system constituted by two coupled two-level atoms (qubits) interacting with a nonlinear generalized cavity field. The nonclassical two-qubit correlation dynamics are investigated using Bures distance entanglement and local quantum Fisher information under the influences of intrinsic decoherence and qubit–qubit interaction. The effects of the superposition of two identical generalized coherent states and the initial coherent field intensity on the generated two-qubit correlations are investigated. Entanglement of sudden death and sudden birth of the Bures distance entanglement as well as the sudden changes in local Fisher information are observed. We show that the robustness, against decoherence, of the generated two-qubit correlations can be controlled by qubit–qubit coupling and the initial coherent cavity states.

## 1. Introduction

Nonclassical correlations (NCs) and quantum entanglement (QE) are substantial as tools for quantum information [1,2,3,4,5]. Therefore, the generation of two-qubit correlated states has been extensively investigated [3,6,7,8]. QE is an important type of NC, but it is not a unique resource in nonclassical correlations [9]. Other types of NCs beyond QE were defined via quantum Fisher information (QFI) [10], local quantum Fisher information (LQFI) [11], quantum discord [12], and other geometrical correlation quantifiers based on skew information [13] and distance norms [14].

Quantum entanglement and purity are recognized as primarily important in developing modern quantum technologies [2,15,16]. The entanglement can be created and preserved between completely separated qubits inside the cavity [17,18,19,20,21,22]. Quantum Fisher information is the most used to describe absolute accuracy in parameter estimation scenarios [23].

Recently, several suggestions have been introduced based on QFI dynamics to demonstrate the importance of quantum entanglement especially for quantum metrology [24] and for parameter estimation precision [25]. On the other hand, Bures distance entanglement (BDE) was used to measure correlations between the parts of a quantum system. The Gaussian entanglement in an identical two-mode Gaussian cavity state was evaluated in terms of its Bures distance with the set of separable Gaussian states [26]. The robustness of the Bures distance discord in comparison with the entanglement against local decoherence was examined [27]. Furthermore, the relationship between the entanglement geometric measure and entanglement distance quantifier was analyzed [28].

Cavity quantum electrodynamics (CQED) focus on the interaction between atoms and a quantum field inside a cavity [29]. The study of CQED began with an emphasis on analyzing basic processes in the interaction of atoms and cavity field based on exploring classical and quantum properties in the presence of decoherence processes [30]. Most of the theoretical and experimental studies concentrate on the single-mode cavity field interacting with single atom [31]. Therefore, multi-mode cavity field interactions may improve the correlation in the quantum system [32]. The high-order nonlinearity of the interactions between atoms and multi-mode cavity were subjected to several applications in quantum information [33,34]. The nonlinear models were widely used to study “exotic” or nonclassical effects such as collapse and revival phenomena [35], quantum filters [36], bistability [37,38], and chaos [39]. Multiple photon processes play an important role in the nonlinear interactions [40,41], which are a convenient resource for quantum information and metrology [24].

Nonlinear coherent states, entangled pair-coherent states [42] and Barut–Girardello nonlinear coherent state (B-GCS) [43] are widely used and applied in physics. There are different approaches to construct them. B-GCS [43] is of particular importance for quantum information [44,45,46]. The construction of the B-GCS was realized for different physical systems as Morse potential [47], Pöschl–Teller potential [48], and charge carriers in anisotropic 2D-Dirac materials immersed in a constant homogeneous magnetic field [49].

In previous investigations of two-qubit dynamics based on new correlation quantifiers, local quantum Fisher information, such as quantum discord and other geometrical correlation quantifiers, were very limited [50], specifically for the case of two qubits interacting with nonlinear coherent cavity fields under the effects of the decoherence/disspation. Therefore, in this work, we study the dynamics of two qubits coupled to a nonlinear cavity in the presence of intrinsic decoherence. An analytical solution for the Milburn equation that describes intrinsic decoherence is obtained when the two qubits are initially in an uncorrelated state and the cavity field is initially prepared in a superposition of two identical generalized Barut–Girardello nonlinear coherent states. In addition, we use new correlation measures, LQFI and BDE, to describe the features of the proposed model such as (1) the ability of the unitary qubit–cavity interactions to generate new types of quantum correlation, (2) the enhancement of two-qubit coupling for the generated correlations, and (3) the robustness of the generated correlation against intrinsic decoherence and the stability of the generated stationary two-qubit state. Finally, sudden death and sudden birth as well as sudden changes during the dynamical behavior of BDE and LQFI, respectively also appear. These features have potential applications in quantum processing, which depend on the generated stable correlation and entanglement [1,2,3], which are reported experimentally [51,52].

The paper is organized as follows: Section 2 introduces the proposed model of the intrinsic decoherence and the nonclassical correlation quantifiers, while the dynamics of the quantum correlations are discussed in Section 3. Finally, the conclusion is presented in Section 4.

## 2. Preliminary

### 2.1. The Physical Model

The problem of the two algebraic systems SU(1,1) and SU(2) in the presence of a Kerr-like medium was studied in [53]. The effect of Stark shift on the interaction between the algebraic system SU(1,1) and one atom was also studied [54]. Therefore, a multiplicity of ${\hat{K}}_{+}$ and ${\hat{K}}_{-}$ generators appears. As an extension of this generalization, we consider the *m*th-order degeneracy of the generators ${\hat{K}}_{+}$ and ${\hat{K}}_{-}$. The considered system is formed by identical two coupled qubits (*A* and *B*) with the same transition frequency ω between their lower and upper states $|0_{i}\rangle$ and $|1_{i}\rangle$ (i=A,B) identified by the energies $\hbar \omega_{0}^{i}$ and $\hbar \omega_{1}^{i}$, respectively. The two qubits interact with the nonlinear generalized cavity field through *m*-photon processes (m=1,2,…). The Hamiltonian can be written as

(1) $$\begin{array}{ccc} \hat{H} & = & \omega_{f}{\hat{K}}_{0}+\omega \left({\hat{\sigma }}_{z}^{A}+{\hat{\sigma }}_{z}^{B}\right)+\sum_{i=A,B}\lambda ({\hat{K}}_{-}^{m}|1_{i}\rangle \langle 0_{i}|+{\hat{K}}_{+}^{m}|0_{i}\rangle \langle 1_{i}|)\\ & & \;+J(|1_{A}0_{B}\rangle \langle 0_{A}1_{B}\left|+\right|0_{A}1_{B}\rangle \langle 1_{A}0_{B}|), \end{array}$$

where $\omega_{f}$ represents the cavity field frequency, λ is the coupling interaction constant between the qubits and the two-mode cavity fields, and *J* represents the dipole–dipole coupling. The SU(1,1) generators satisfy the following:

(2) $$\begin{array}{ccc} \left[{\hat{K}}_{0},{\hat{K}}_{\pm }\right] & = & \pm {\hat{K}}_{\pm },\;\left[{\hat{K}}_{-},{\hat{K}}_{+}\right]=2{\hat{K}}_{0}, \end{array}$$

(3) $$\begin{array}{ccc} {\hat{K}}_{+}{\hat{K}}_{-} & = & {\hat{K}}_{0}^{2}-\left({\hat{K}}_{0}+{\hat{K}}^{2}\right), \end{array}$$

(4) $$\begin{array}{ccc} {\hat{K}}^{2} & = & {\hat{K}}_{0}^{2}-\frac{1}{2}\left({\hat{K}}_{+}{\hat{K}}_{-}+{\hat{K}}_{-}{\hat{K}}_{+}\right)=k\left(k-1\right)\hat{I}. \end{array}$$

The ${\hat{K}}^{2}=k\left(k-1\right)\hat{I}$ is the Casimir operator, whereas *k* represents the Bargmann number. For the complete orthonormal basis, {|n,k〉(n=0,1,2,...;k=const.)},

(5) $$\begin{array}{ccc} K_{+}^{m}|n,k\rangle & = & \Lambda_{n,m}\;\;|n+m,k\rangle , \end{array}$$

(6) $$\begin{array}{ccc} K_{-}^{m}|n,k\rangle & = & \Lambda_{n-1,m}\;\;|n-m,k\rangle , \end{array}$$

(7) $$\begin{array}{ccc} \Lambda_{n,m} & = & \sqrt{\frac{(m+n)!(m+n+2k-1)!}{n!(n+2k-1)!}} \end{array}$$

There are physical Hamiltonians system that could be converted to the above Hamiltonian using Lie algebraic operators. For example, (1) let us first consider the case where the qubit interacts with the cavity field containing a two-mode nondegenerate parametric amplifier. In this case, the SU(1,1) generators [55,56], ${\hat{K}}_{\pm }$, and ${\hat{K}}_{0}$ are introduced as follows:

(8) $$\begin{array}{ccc} {\hat{K}}_{-} & = & \frac{1}{2}{\hat{a}}_{1}{\hat{a}}_{2}={\hat{K^{†}}}_{+}, \end{array}$$

(9) $$\begin{array}{ccc} {\hat{K}}_{0} & = & \frac{1}{4}\left({\hat{a}}_{1}^{†}{\hat{a}}_{1}+{\hat{a}}_{2}^{†}{\hat{a}}_{2}+\hat{I}\right), \end{array}$$

where ${\hat{a}}_{i}^{†}$ and ${\hat{a}}_{i}$ are the creation and annihilation operators of the field inside the cavity. (2) We consider also a second case where the qubit interacts with a nonlinear (*f*-deformed) cavity field, ${\hat{K}}_{-}=\hat{a}f\left({\hat{a}}^{†}\hat{a}\right)={\left({\hat{K}}_{+}\right)}^{†}$, where $f({\hat{a}}^{†}\hat{a})$ represents the hermitian operator-valued functions responsible for intensity-dependent coupling. (3) The third considered case is for the qubit interacting with a two-photon process. In this case,

(10) $$\begin{array}{ccc} {\hat{K}}_{-} & = & \frac{1}{2}{\hat{a}}^{2}={\hat{K^{†}}}_{+},\;{\hat{K}}_{0}=\frac{1}{2}\left({\hat{a}}^{†}\hat{a}+\hat{I}\right). \end{array}$$

To study the decoherence effect on the cavity–qubit system dynamics, we use the intrinsic decoherence (ID) model [57], which describes the decoherence effect as the system evolves. The Milburn equation governs the dynamics of this system:

(11) $$\begin{array}{ccc} \frac{d}{dt}\rho \left(t\right) & = & \hat{L}\rho \left(t\right),\;\;\;\hat{L}*=-i\left[\hat{H},*\right]-\frac{\gamma }{2}\left[\hat{H},\left[\hat{H},*\right]\right], \end{array}$$

where $\hat{H}$ is the physical considered Hamiltonian system of Equation (1), γ represents the intrinsic parameter, and the Milburn equation is reduced to the Schrödinger equation if γ=0. The condition of intrinsic decoherence is that, for a short time, the cavity–qubit system evolves by stochastic sequences of identical unitary transformations rather than by continuous unitary evolutions [57].

The two two-level systems are initially in the upper state, i.e., ${\hat{\rho }}^{A}\left(0\right)=|1_{A}1_{B}\rangle \langle 1_{A}1_{B}|$, while the initial cavity density matrix is described by a superposition of two identical generalized Barut–Girardello nonlinear coherent states (B-GCS) with π-angle, |α,k〉 and |−α,k〉 [43,58], as follows:

(12) $$\begin{array}{c} \;\;\;\;\;\;{\hat{\rho }}^{f}\left(0\right)=\frac{1}{A}{(|\alpha ,k\rangle +r|-\alpha ,k\rangle )(|\alpha ,k\rangle +r|-\alpha ,k\rangle )}^{†}, \end{array}$$

where *A* designs the normalization factor. The cavity is initially in the Barut–Girardello nonlinear coherent state for r=0, while it is in even B-GCS for r=1. For the basis {|n,k〉(n=0,1,2,⋯;k=const.)}, the B-GCS is defined as follows [43]:

(13) $$\begin{array}{ccc} |\alpha ,k\rangle & = & \sum_{m=0}^{\infty }F_{m}|m,k\rangle ,\;F_{m}^{2}=\frac{{\left|\alpha \right|}^{2k-1}\alpha^{2m}}{n!\left(m+2k-1\right)I_{2k-1}\left(2|\alpha |\right)}, \end{array}$$

where α represents the initial intensity Barut–Girardello nonlinear coherent cavity and $I_{\nu }\left(x\right)$ is the modified Bessel function. The even and odd coherent states are special cases for the Barut–Girardello coherent states when $k=\frac{1}{4}$ and $k=\frac{3}{4}$, respectively. Another special case from the B-GCS is the nonlinear coherent state $|\alpha ,\frac{1}{2}\rangle$ ($k=\frac{1}{2}$). Consequently, the initial state of the cavity–qubit system is given by

(14) $$\begin{array}{c} \hat{\rho }\left(0\right)={\hat{\rho }}^{f}\left(0\right)⊗|1_{A}1_{B}\rangle \langle 1_{A}1_{B}|. \end{array}$$

By using Equation (11) and the initial state of Equation (14), in the space eigenstates $\left\{\;|D_{i}^{n}\rangle \;\right\}$ of the Hamiltonian of Equation (1), the qubit–cavity density matrix is given by

(15) $$\begin{array}{ccc} \hat{\rho }\left(t\right) & = & \sum_{m,n=0}\sum_{r=1,3,4}F_{m}F_{n}^{*}\{C_{r1}^{m}C_{11}^{n}H_{r1}|D_{r}^{m}\rangle \langle D_{1}^{n}|\\ & & +C_{r1}^{m}C_{31}^{n}H_{r3}|D_{r}^{m}\rangle \langle D_{3}^{n}|+C_{r1}^{m}C_{41}^{n}H_{r4}|D_{r}^{m}\rangle \langle D_{4}^{n}|\}, \end{array}$$

where

$$H_{r,s}=\left[\cos\left(V_{r}^{m}-V_{s}^{n}\right)t-i\sin\left(V_{r}^{m}-V_{s}^{n}\right)t\right]T_{\mathrm{erm}}^{rs}.$$

where $T_{\mathrm{erm}}^{rs}=e^{-\frac{\gamma }{2}{\left(V_{r}^{m}-V_{s}^{n}\right)}^{2}t}\left(r,s=1,3,4\right)$ represents the decoherence term. $C_{rj}^{n}$ is the coefficients of the eigenstates $\left\{\;|D_{r}^{n}\rangle \;\right\}$ of the Hamiltonian of Equation (1) with the corresponding eigenvalues $V_{r}^{n}\;\left(r=1-4\right)$.

Based on the cavity number state |n〉 (for n=0,1,2,3,...) and the two-qubit system space state $\{|1_{A}1_{B}\rangle ,|1_{A}0_{B}\rangle ,|0_{A}1_{B}\rangle ,|0_{A}0_{B}\rangle$, the space eigenstates SU(1,1)–SU(2) system is identified by $\{|\varpi_{1}\rangle =|n,1_{A}1_{B}\rangle ,|\varpi_{2}\rangle =|n+m,1_{A}0_{B}\rangle ,|\varpi_{3}\rangle =|n+m,0_{A}1_{B}\rangle ,|\varpi_{4}\rangle =|n+2m,0_{A}0_{B}\rangle \}$ and

(16) $$\begin{array}{ccc} |D_{r}^{n}\rangle & = & \sum_{j=1}^{4}C_{rj}^{n}|\varpi_{j}\rangle . \end{array}$$

Therefore, $|D_{r}^{n}\rangle$ satisfies

(17) $$\begin{array}{ccc} \hat{H}|D_{r}^{n}\rangle & = & V_{r}^{n}|D_{r}^{n}\rangle \;\left(r=1-4\right), \end{array}$$

where the corresponding eigenvalues $V_{r}^{n}$ are given by

(18) $$\begin{array}{ccc} V_{1}^{n} & = & \omega (m+n+k),\\ V_{2}^{n} & = & \omega (m+n+k)-J,\\ V_{3}^{n} & = & \omega \left(m+n+k\right)+\frac{1}{2}J\\ & & \;-\frac{1}{2}\sqrt{J^{2}+8\lambda^{2}\left(\Lambda_{n,m}^{2}+\Lambda_{n,m}^{2}\right)},\\ V_{4}^{n} & = & \omega (m+n+k)\\ & & \;+\frac{1}{2}J+\frac{1}{2}\sqrt{J^{2}+8\lambda^{2}\left(\Lambda_{n,m}^{2}+\Lambda_{n,m}^{2}\right)}. \end{array}$$

After obtaining the general solution $\hat{\rho }\left(t\right)$, we consider the case of m=1 and the resonance case ($\omega =\omega_{f}$) to investigate some quantum correlations under the effects of unitary interaction, intrinsic noise, and qubit–qubit coupling. The case m=1 corresponds to a one-photon process for the case of a nonlinear (*f*-deformed) cavity field and a one-photon process when the cavity field contains a two-mode parametric amplifier.

### 2.2. Nonclassical Correlation Quantifiers

Nonclassical correlations are important resources for quantum information [59]. The two-qubit nonclassical correlations are investigated by using local quantum Fisher information and Bures distance entanglement.

### 2.3. Local Quantum Fisher Information (LQFI)

Recently, local quantum Fisher information was introduced as an important quantifier of NCCs, which is defined as the minimum quantum Fisher information (LQFI). Let us consider a given bipartite quantum state (with parts *A* and *B*) $\rho^{AB}$ in the Hilbert space $H=H_{A}⊗H_{B}$ ($H_{l},l=A,B)$, which is the local Hamiltonian acting on the *l*-part. The LQFI of $\rho^{AB}$, associated with the local evolution generated by a Hermitian operator $I_{A}⊗H_{B}$ with the *A*-part identity operator $I_{A}$, can be written as follows [60]

(19) $$\begin{array}{ccc} F( & \rho^{AB} & ,H_{B})\\ & = & 4\;\;\;\;\;\;\;\;\sum_{\pi_{m}+\pi_{n}>0}\frac{{\left(\pi_{m}-\pi_{n}\right)}^{2}}{\pi_{m}+\pi_{n}}|\langle \psi_{m}|I_{A}⊗H_{B}|\psi_{n}{\rangle |}^{2}. \end{array}$$

where $\left\{\pi_{m}\right\}$ and $\left\{|\psi_{m}\rangle \right\}$ represent the eigenvalues and the eigenstates of the bipartite state $\rho^{AB}$, where $\rho^{AB}=\sum_{m}\pi_{m}|\psi_{m}\rangle \langle \psi_{m}|$, with $\pi_{m}\geq 0$ and $\sum_{m}\pi_{m}=1$. Therefore, Equation (19) can be rewritten as

(20) $$\begin{array}{ccc} F(\rho^{AB},H_{B}) & = & 4\mathrm{Tr}\{\rho^{AB}H_{B}^{2}\}\\ & - & \sum_{m,n}\frac{8\pi_{m}\pi_{n}}{\pi_{m}+\pi_{n}}|\langle \psi_{m}|I_{A}⊗H_{B}|\psi_{n}{\rangle |}^{2}. \end{array}$$

If the bipartite quantum state $\rho^{AB}$ is for the case of the two qubits, then the nonclassical correlation can be quantified by the minimum QFI over all local Hamiltonians $H_{B}$ of a fixed spectral class, which are reduced to $H_{B}=\vec{r}.\vec{\sigma }$, where $|\vec{r}|=1$ and $\vec{\sigma }=\sigma_{x},\sigma_{y},\sigma_{z}$ is the Pauli vector. The local quantum Fisher information is given by the following expression [11,61]:

(21) $$\begin{array}{ccc} F(t) & = & 1-\pi_{W}^{\max}, \end{array}$$

where $\pi_{W}^{\max}$ represents the highest eigenvalue of the real 3×3 symmetric matrix $W[w_{ij}]$ with elements $w_{ij}$,

$$\begin{array}{ccc} w_{ij} & = & \sum_{m,n=1}^{4}\frac{2\pi_{m}\pi_{n}}{\pi_{m}+\pi_{n}}\langle \psi_{m}\left|U\right|\psi_{n}\rangle \langle \psi_{n}|U^{†}|\psi_{m}\rangle , \end{array}$$

and $U=I_{A}⊗\sigma_{B}^{i}$. $\left\{\pi_{m}\right\}$ and $\left\{|\psi_{m}\rangle \right\}$ are the eigenvalues and the eigenstates of the two-qubit reduced density matrix $\rho^{AB}\left(t\right)$, which are determined numerically. In Equation (15), the two-qubit reduced density matrix $\rho^{AB}\left(t\right)$ is defined as

(22) $$\begin{array}{ccc} \rho^{AB}\left(t\right) & = & {\mathrm{Tr}}_{C}\left\{\hat{\rho }\left(t\right)\right\}. \end{array}$$

where the operation ${\mathrm{Tr}}_{C}$ traces the cavity states.

### 2.4. Bures Distance Entanglement

The measure of the BDE [28] depends on the concurrence [62], which is defined by

(23) $$\begin{array}{c} B\left(t\right)=\sqrt{2-\sqrt{2+2\sqrt{1-C{\left(t\right)}^{2}}}}, \end{array}$$

where C(t) is the concurrence function,

(24) $$\begin{array}{c} C\left(t\right)=\max\left\{0,\sqrt{\lambda_{1}}-\sqrt{\lambda_{2}}-\sqrt{\lambda_{3}}-\sqrt{\lambda_{4}}\;\right\}, \end{array}$$

where $\lambda_{1}\geq \lambda_{2}\geq \lambda_{3}\geq \lambda_{4}$ designate the enginevalues of the matrix: $R=\rho^{AB}\left(\sigma_{y}⊗\sigma_{y}\right)\rho^{AB*}\left(\sigma_{y}⊗\sigma_{y}\right)$. The value of B(t) is bounded by two values: zero, which represents the case of unentangled states, and $\sqrt{2-\sqrt{2}}$ for a maximal entangled state.

## 3. Nonclassical Correlation Dynamics

**Figure 1:**
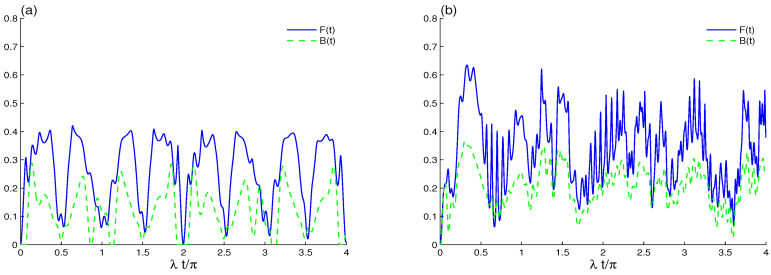
Local quantum Fisher information (LQFI) and Bures distance entanglement (BDE) dynamics of the uncorrelated two-qubit state when the initial cavity field is in the B-GCS (r=0) with $k=\frac{1}{2}$ and the initial coherent intensity ${\left|\alpha \right|}^{2}=16$ in the absence of the intrinsic decoherence. The qubit–qubit coupling effect is shown with different values J=0 in (**a**) and J=20λ in (**b**).

**Figure 2:**
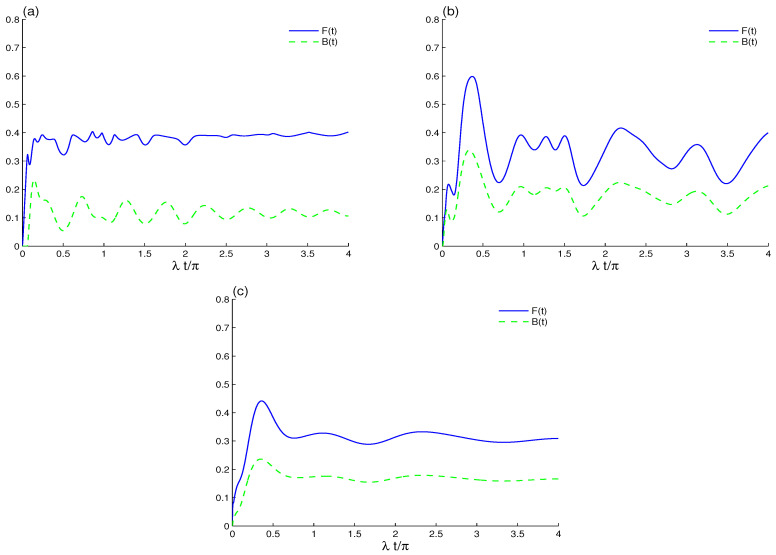
LQFI and BDE dynamics when the initial cavity field is in the B-GCS with $k=\frac{1}{2}$ and the initial coherent intensity ${\left|\alpha \right|}^{2}=16$ in the presence of the intrinsic decoherence effect γ=0.01λ in (**a**,**b**), γ=0.1λ in (**c**). The qubit–qubit coupling effect is shown with different values J=0 in (**a**) and J=20λ in (**b**).

**Figure 3:**
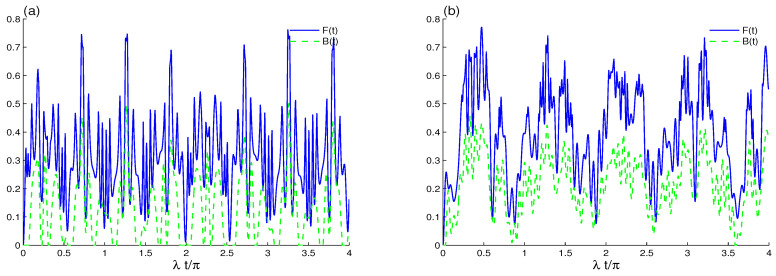
LQFI and BDE dynamics in the case where the initial cavity field is in the even B-GCS (r=1) with the initial coherent intensity ${\left|\alpha \right|}^{2}=16$ and the intrinsic decoherence is absent. With different values of qubit–qubit coupling, J=0 in (**a**) and J=20λ in (**b**).

**Figure 4:**
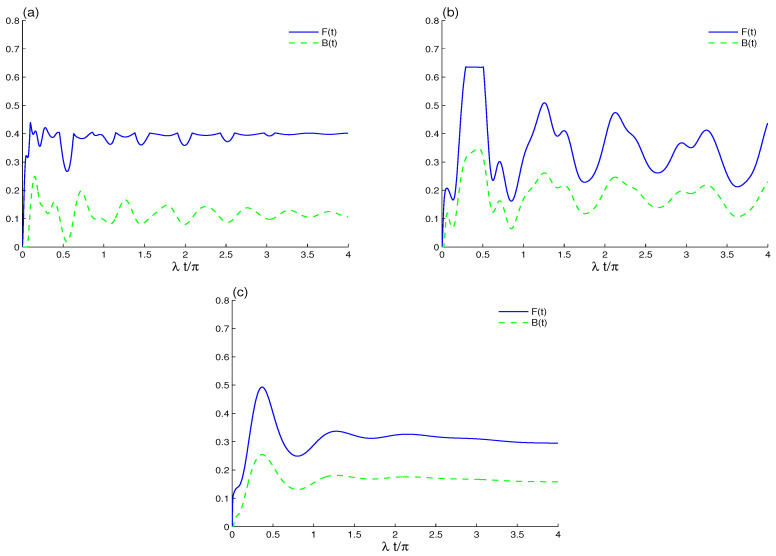
LQFI and BDE dynamics when the initial cavity field is in the even B-GCS with $k=\frac{1}{2}$ and the initial coherent intensity ${\left|\alpha \right|}^{2}=16$ in the presence of the intrinsic decoherence effect γ=0.01λ in (a,b), γ=0.1λ in (**c**). The qubit–qubit coupling effect is shown with different values J=0 in (**a**) and J=20λ in (**b**).

**Figure 5:**
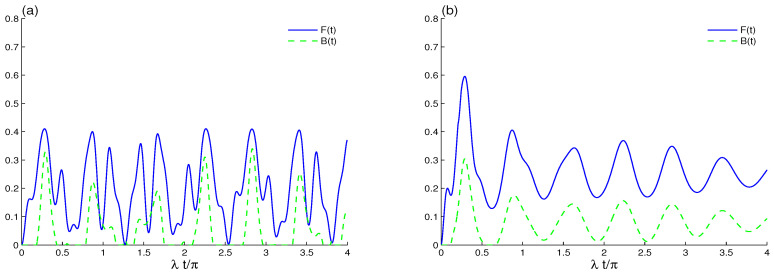
LQFI and BDE dynamics when the initial cavity field is in the B-GCS with $k=\frac{1}{2}$, small initial coherent intensity ${\left|\alpha \right|}^{2}=1$, and the qubit–qubit coupling effect is absent. The decoherence effect is shown with different values γ=0.0 in (**a**) and γ=0.01λ in (**b**).

**Figure 6:**
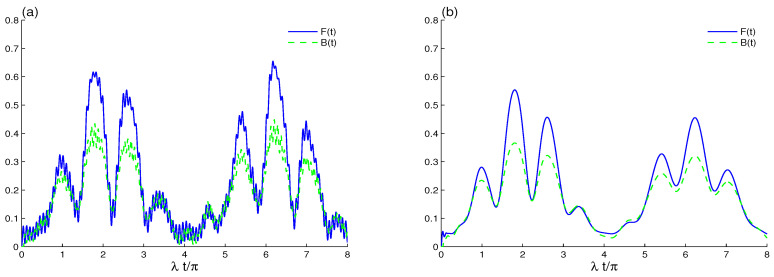
LQFI and BDE dynamics when the initial cavity field is in the B-GCS with $k=\frac{1}{2}$, small initial coherent intensity ${\left|\alpha \right|}^{2}=1$ in the presence of the qubit–qubit coupling effect J=20λ. The decoherence effect is shown with different values γ=0.0 in (**a**) and γ=0.01λ in (**b**).

In this section, we are interested in studying the effects of qubit–qubit coupling (Figure 1), intrinsic decoherence (Figure 2), and the initial coherent cavity field (Figure 3 and Figure 4). Finally, the effect of the intensity coherent field ${\left|\alpha \right|}^{2}$ is presented in Figure 5 and Figure 6.

For the generalized Barut–Girardello nonlinear coherent state r=0, we estimated the correlation between the two qubits by the LQFI and BDE, respectively, in Figure 1 where the intrinsic decoherence is absent. We note that the oscillatory behaviors of the correlation functions F(t) and B(t) have different amplitudes and frequencies. Figure 1a shows the dynamics of the LQFI and BDE for the case where there is no decoherence γ=0 and no direct qubit interaction J=0 with m=1. The evolution in this case is unitary for the two qubits that interact with the generalized Barut–Girardello nonlinear coherent cavity field via the interaction term: $\lambda ({\hat{K}}_{-}|1_{i}\rangle \langle 0_{i}|+{\hat{K}}_{+}|0_{i}\rangle \langle 1_{i}|)$, where the operators ${\hat{K}}_{\pm }$ might be close to a two-mode cavity field operator. Note that the LQFI correlation is periodically generated with 2π-period. In each period, the ability of the cavity–qubit interactions to induce the LQFI correlation depends on time. In contrast, the irregular oscillatory behavior of the BDE vanishes suddenly for short intervals and then stands up suddenly. This process shows that the phenomena of sudden death (i.e., abrupt disappearance of the Bures distance entanglement at a finite time) and sudden birth (the Bures distance entanglement sudden revival) of the Bures distance entanglement can be achieved; see Figure 1a. In the intervals between the sudden death and the sudden birth, the generated two-qubit entanglement can spread throughout the qubit–cavity system. This means that the transitions of the Bures distance entanglement are intimately related to sudden death and sudden birth of entanglement. The distribution of the entanglement, including sudden death and sudden birth phenomena of the entanglement, has been theoretically [63,64,65] and experimentally reported [66,67].

Figure 1b illustrates that qubit–qubit coupling generates the LQFI correlation and the BDE. We find that the increase in qubit–qubit coupling leads to enhancement of the amplitudes and frequencies of the nonclassical correlations. The phenomenon sudden death and sudden birth of the BDE and the initial disentanglement interval disappear completely only in the presence of qubit–qubit coupling. It is difficult to transfer qubit–qubit entanglement to the cavity fields. Qubit–qubit coupling plays an important role in generating qubit–qubit entanglement. This qubit-qubit coupling effect is expected from the Hamiltonian of Equation (1), where the interaction terms involving the two-qubit operators naturally turn a superable state of the type $|1_{A}0_{B}\rangle$ into a state $(\alpha |1_{A}0_{B}\rangle +\beta |0_{A}1_{B}{\rangle ),\;|\alpha |}^{2}+{\left|\beta \right|}^{2}=1$, which could be close to the maximally entangled state. We deduce that the generated qubit–qubit correlations of LQFI and BDE can be enhanced by increasing the qubit–qubit coupling.

In Figure 2 we display the influence of intrinsic decoherence on the qubit–qubit correlation. In the absence of qubit–qubit coupling, the LQFI oscillations are decayed, while its correlation grows to a stationary value. In this case, two-qubit LQFI correlation is still time-dependent; see Figure 2a. The dashed curve of Figure 2a shows that the amplitudes of BDE are reduced and that the two-qubit state quickly reaches a quasi-steady entangled state. The stability of the stationary LQFI is more pronounced than that of Bures distance entanglement. The entangled two-qubit state and the stability of the LQFI correlation as well as the BDE depend on intrinsic decoherence. The entanglement sudden death and sudden birth of the Bures distance disappears due to the intrinsic decoherence effect, which leads to increased minima of the Bures distance function to a stationary value.

In Figure 2b, we combine the effects of ID and qubit–qubit coupling. A smooth growth arises in the correlations, and both measures take a long time to reach a steady state after considering coupling to the cavity. Through these results, we deduce that qubit–qubit coupling leads to an increase in LQFI and BDE nonclassical correlations. The stability of LQFI and BDE is delayed. The generated qubit–qubit correlations via LQFI and BDE are more robust against decoherence in the presence of qubit–qubit coupling. Figure 2c shows that, to observe the stationary qubit–qubit state with qubit–qubit coupling, the parameter of the intrinsic decoherence must be increased, γ=0.1λ.

Figure 3 and Figure 4 display the time evolution of Bures distance entanglement and the local quantum Fisher information for the even coherent cavity field with the same parameter values as in Figure 1 (coherent state). From Figure 3a, we observe that the maximum values of the LQFI and BDE functions increase compared to the case of the coherent state. Moreover, setting the cavity in the even coherent state enhances generation of the nonclassical correlation between the two qubits. The sudden birth and sudden death phenomena of Bures distance entanglement are achieved during several time intervals.

The results of Figure 3b confirm the effects of qubit–qubit coupling. The intensity of the oscillations of F(t) decreases while its amplitudes increase after adding qubit–qubit coupling to the cavity. In addition, a strong BDE between the two qubits is present. The phenomena of sudden death and sudden birth of the BDE are almost disappeared. In the case of the even coherent state, the qubit–qubit coupling enhances remarkably the correlations presented by Bures distance entanglement and local quantum Fisher information.

After considering decoherence, LQFI and BDE correlations are generated, and after a short time, their amplitudes are substantially reduced to constant values; see Figure 4a. The correlation identified by the LQFI quantifier is more stable and robust against decoherence than that of Bures distance entanglement. The phenomenon of sudden changes occurs (which was observed experimentally [51] and analytically in several systems [68,69,70]) only during the dynamic behavior of the LQFI function F(t). A strong correlation arises between the two qubits. Therefore, coupling between the qubits and the cavity field can conserve or enhance the correlations which resist to decoherence. Figure 4b confirms that the qubit–qubit interaction enhances the qubit–qubit correlation. Furthermore the phenomenon of sudden changes in the LQFI correlation is clearly observed. Figure 4c shows that, after adding qubit–qubit coupling, the phenomenon of sudden changes and the stationary correlations depend on the increase in the intrinsic decoherence.

Figure 5 and Figure 6 display the effect of the intensity coherent field ${\left|\alpha \right|}^{2}$ for a cavity initially in an even generalized coherent state. The generated nonclassical correlation of the LQFI has regular oscillatory behavior, while the Bures distance entanglement has irregular oscillations that are reduced during most of the interaction period; see Figure 5. By comparing the results of the two cases of the large and small intensity coherent values, we find that, with small intensity coherent value, the LQFI correlation is enhanced while the Bures distance two-qubit entanglement is reduced. The sudden death and sudden birth phenomena of the two-qubit entanglement are observed. The disentanglement time intervals are very large compared to previous cases. Note that, in these time intervals, the disentangled two-qubit state has a partial LQFI correlation. For a small intensity coherent field, the effect of the intrinsic decoherence is weakened. The dynamics of the LQFI is more robust than the BDE.

Figure 6 shows the effect of the two-qubit coupling with a small intensity coherent field. A symmetric relation appears between the correlation functions. Moreover, the maximum values of the quantum correlations increase while their minimum values are shifted up. In other words, nonclassical correlations can be enhanced and are more robust against ID due to the intensity coherent field.

## 4. Conclusions

In this paper, we considered a system constituted by two coupled qubits interacting with a nonlinear generalized SU(1,1) cavity field. An analytical solution of the intrinsic decoherence model for the considered system was obtained when the SU(1,1) cavity field initially had a superposition of two identical generalized coherent states. The two-qubit nonclassical correlation dynamics were investigated by using Bures distance entanglement and local quantum Fisher information under the influence of intrinsic decoherence and qubit–qubit coupling. In a generalized coherent state, two-qubit nonclassical correlations were generated and improved in the presence of qubit–qubit coupling. The phenomena of sudden death and sudden birth were observed in the Bures distance entanglement dynamics. The two-qubit nonclassical correlations can be enhanced for the even generalized coherent cavity field. They are more pronounced after considering qubit–qubit coupling for the qubit–cavity interaction. The generated correlations are stabilized by intrinsic decoherence. The nonclassical correlations can be enhanced and are more robust against decoherence due to the intensity coherent field.

## Data Availability

Not applicable.
